# PP2A in LepR+ mesenchymal stem cells contributes to embryonic and postnatal endochondral ossification through Runx2 dephosphorylation

**DOI:** 10.1038/s42003-021-02175-1

**Published:** 2021-06-02

**Authors:** Yu-Ting Yen, May Chien, Pei-Yi Wu, Shih-Chieh Hung

**Affiliations:** 1 Drug Development Center, Institute of New Drug Development, China Medical University, Taichung, Taiwan; 2 Integrative Stem Cell Center, China Medical University Hospital, Taichung, Taiwan; 3 Department of Orthopaedics, China Medical University Hospital, Taichung, Taiwan

**Keywords:** Bone development, Mesenchymal stem cells

## Abstract

It has not been well studied which cells and related mechanisms contribute to endochondral ossification. Here, we fate mapped the leptin receptor-expressing (LepR^+^) mesenchymal stem cells (MSCs) in different embryonic and adult extremities using *Lepr-cre; tdTomato* mice and investigated the underling mechanism using *Lepr-cre; Ppp2r1a*^*fl/fl*^ mice. Tomato^+^ cells appear in the primary and secondary ossification centers and express the hypertrophic markers. Ppp2r1a deletion in LepR^+^ MSCs reduces the expression of Runx2, Osterix, alkaline phosphatase, collagen X, and MMP13, but increases that of the mature adipocyte marker perilipin, thereby reducing trabecular bone density and enhancing fat content. Mechanistically, PP2A dephosphorylates Runx2 and BRD4, thereby playing a major role in positively and negatively regulating osteogenesis and adipogenesis, respectively. Our data identify LepR^+^ MSC as the cell origin of endochondral ossification during embryonic and postnatal bone growth and suggest that PP2A is a therapeutic target in the treatment of dysregulated bone formation.

## Abstract (web summary)

Yen et al use tissue-specific PP2A knockout mice to show that PP2A in LepR positive mesenchymal stem cells positively regulates endochondral ossification. They find that PP2A dephosphorylates Runx2 and BRD4, thereby playing a major role in positively and negatively regulating osteogenesis and adipogenesis, respectively.

## Introduction

Skeletal growth during limb development is divided into several stages. In embryo development, each upper and lower limb initially develops as a small bulge called a limb bud, which is formed by mesenchyme, a type of embryonic tissue that can differentiate into many types of tissues, including bone or muscle tissue^1^. The mesenchyme within the limb bud then differentiates into cartilage, which forms models for future bones. By embryonic day (E)15.5 in mice, the primary ossification centers^2^ (POCs) have developed in the diaphysis of long bones in the limbs, initiating the process that converts the cartilage model into bone^3^. A secondary ossification center (SOC) will appear in each epiphysis of the long bones at a later time, usually starting before postnatal day (P)10^3^. The epiphyseal plate, a layer of growing hyaline cartilage, is located between the diaphysis and each epiphysis. It continues to grow and is responsible for the lengthening of the long bones.

There are two modes of bone formation. Intramembranous ossification is involved in the formation of the flat bones, such as the skull, mandible and clavicle, in which mesenchymal cells condense and directly differentiate into osteoblasts to deposit bone matrix. Endochondral ossification occurs during the formation of the long bones and encompasses the processes of POC, SOC, and epiphyseal growth, where a cartilage mold is formed first and subsequently replaced by bone tissue^1^. There are many pathways involved in the process of endochondral ossification, including the hedgehog, Wnt, bone morphogenetic protein (BMP), and Notch pathways^4^.

Runt-related transcription factor 2 (Runx2), also known as Cbfa1, PEBP2A1, and AML3, is involved in the activation of genes encoding osteoblast- and chondrocyte-specific proteins^5,6^. Genetic studies have shown that Osterix^7^, collagen X^8^, and MMP13^9^ are important Runx2 downstream genes, which are involved in endochondral ossification. Runx2 activity is regulated by transcriptional and post-transcriptional mechanisms. Phosphorylation of Runx2 by cyclin D1-cyclin dependent kinase 4 (Cdk4) mediates Runx2 ubiquitination and degradation^10^, thus allowing the regulation of Runx2 activity to be coordinated by the cell cycle machinery. However, the molecular mechanisms regulating the post-translational modification of Runx2 during endochondral ossification have not been fully defined.

Bromodomain-containing protein 4 (BRD4) is a member of the bromodomain and extraterminal domain (BET) protein family, and acts as an epigenetic reader by binding to acetylated histones^11^. Lee and colleagues demonstrated that BRD4 binds to active enhancers through enhancer epigenetic writers MLL3/4 during adipogenesis, which facilitates the recruitment of positive transcription elongation factor (p-TEFb), RNA polymerase II (Pol II) and transcription factor II D (TFIID)^12^. BRD4 knockout models showed a decrease in adipose tissue in vivo, and the mice displayed an abnormal hunched posture. The disruption of BRD4 in BRD4 knockout cell lines inhibits PPARγ expression and suppresses adipogenesis^13^. This indicates that BRD4 is an important factor for adipogenesis both in vivo^12^ and in vitro^13^. Furthermore, BRD4 is an epigenetic regulator and transcription cofactor whose phosphorylation by CK2 and dephosphorylation by PP2A modulates its function in chromatin targeting and factor recruitment^14^.

Serine/threonine protein phosphatase 2 A (PP2A) participates in regulating many important physiological processes such as growth, apoptosis, and signal transduction. PP2A is requisite for the function of regulatory T (Treg) cells^15^. Treg cells exhibit high PP2A activity in comparison to conventional T cells, and Treg cell-specific ablation of the PP2A complex leads to a severe, multi-organ, lymphoproliferative autoimmune disorder. Knockdown of the α-isoform of the PP2A catalytic subunit (PP2A Cα) in MC3T3-E1 cells enhances bone formation by upregulating the bone-specific transcription factor Osterix^16^. Catechin stimulates osteogenesis by enhancing PP2A activity in human mesenchymal stem cells (MSCs)^17^. These data suggest that the role of PP2A in bone formation is still up for debate. The role of PP2A Cα in bone formation has been examined in vivo by generating transgenic mice expressing the dominant negative form of PP2A Cα. These transgenic mice exhibited an increase in body weight, cortical bone mineral density, and cortical bone thickness^18^. However, the PP2A-associated target substrate has not yet been identified, and the role of PP2A in endochondral ossification has not been examined.

Various skeletal stem cells have contributed to skeletal growth in limb development. Recently, cell-lineage analysis revealed that parathyroid hormone-related protein (PTHrP)-positive chondrocytes within the resting zone of the postnatal growth plate continue to form columnar chondrocytes, undergo hypertrophy, and become osteoblasts and marrow stromal cells beneath the growth plate^19^. Studies based on single-cell RNA sequencing analysis revealed that collagen 2.3 positive (Col2.3^+^) cells might undergo osteogenic transdifferentiation by expressing osteogenic and chondrocyte-specific genes, such as Col10a1^20^. A similar approach revealed that leptin receptor-positive (LepR^+^) MSCs^21^ produce hematopoietic stem cell regulators and partition into subsets spanning a differentiation continuum comprising distinct osteoblast differentiation trajectories^22^. However, its involvement in limb development from embryo to adult has not been well studied.

In the current study, we demonstrate the involvement of LepR^+^ MSCs in embryonic and postnatal bone growth. We further explore the roles of PP2A and its downstream signaling pathways in endochondral ossification and adipogenesis, mediated by LepR^+^ MSCs through the conditional deletion of *Ppp2r1a*, the gene encoding the predominant PP2A scaffold- α isoform subunit^23^, in LepR-expressing cells. Using tissue-specific PP2A knockout (KO) mice, we provide in vitro and in vivo evidence that PP2A positively regulates endochondral ossification by directly associating with and stabilizing Runx2, and negatively regulates adipogenesis through BRD4 dephosphorylation and inactivation, thereby downregulating adipogenic transcription factors.

## Results

### Contribution of LepR^+^ MSCs to POCs and SOCs but not to growth plates of long bones

**Fig. 1:**
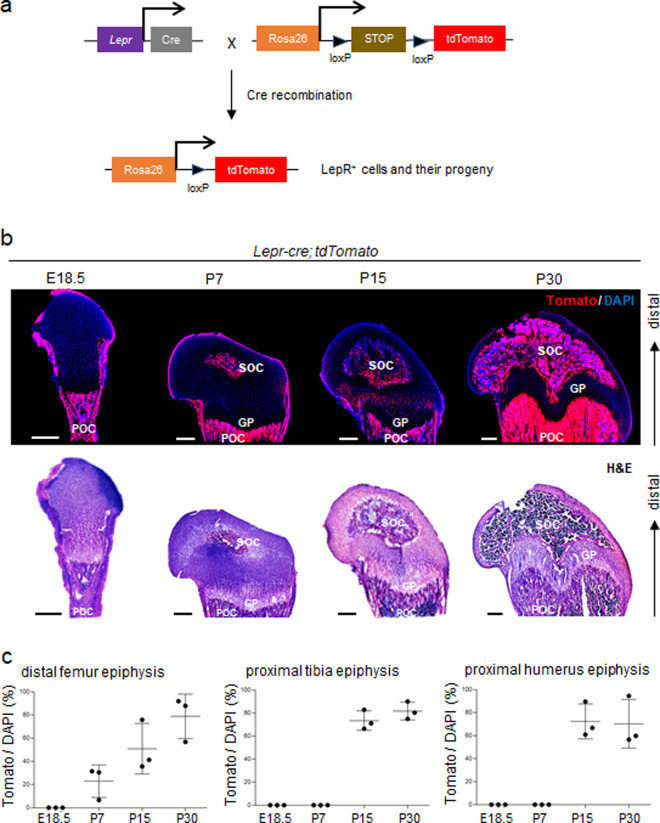
Fate mapping of LepR^+^ MSCs during embryonic and postnatal growth. **a** Illustration of in vivo lineage-tracing experiments with *Lepr-cre; tdTomato* mice. **b** The POC and SOC in the distal femur of *Lepr-cre; tdTomato* mice were histologically examined on E18.5, P7, P15 and P30. Representative pictures showing the appearance of Tomato^+^ cells at indicated stages. Tomato^+^ (red) and DAPI (blue). The corresponding H&E stain are right below. GP = Growth plate; POC = Primary ossification center; SOC = Secondary ossification center. **c** The percentages of Tomato^+^ cells in SOC at indicated stages and regions. Scale bar, 300 μm. *n* = 3 mice. Data are mean ± s.d.

To provide insights into the contribution of LepR^+^ MSCs to limb development, we linage-traced LepR^+^ MSCs in vivo using *Lepr-cre; tdTomato* mice (Fig. 1a). At E18.5, Tomato^+^ cells were mainly observed in the diaphyses of the femur (Fig. 1b), tibia (Fig. S1a) and humerus (Fig. S1b) in *Lepr-cre; tdTomato* mice (which correspond to the POCs), and we observed no contribution of these cells to epiphyses and growth plates in any of these long bones (Fig. 1b). By P7 in the distal femur (Fig. 1b) and P15 in the proximal tibia (Fig. S1a) and proximal humerus (Fig. S1b), there was a sharp increase in the number of Tomato^+^ cells in the center of epiphyses, corresponding to SOCs. Over the course of time up to P30, Tomato^+^ cells expanded to almost all of the epiphyses (Fig. 1c). These data suggest that LepR^+^ MSCs contribute to POCs and SOCs but not to the growth plates of long bones.

### Involvement of LepR^+^ MSCs in the endochondral ossification process

**Fig. 2:**
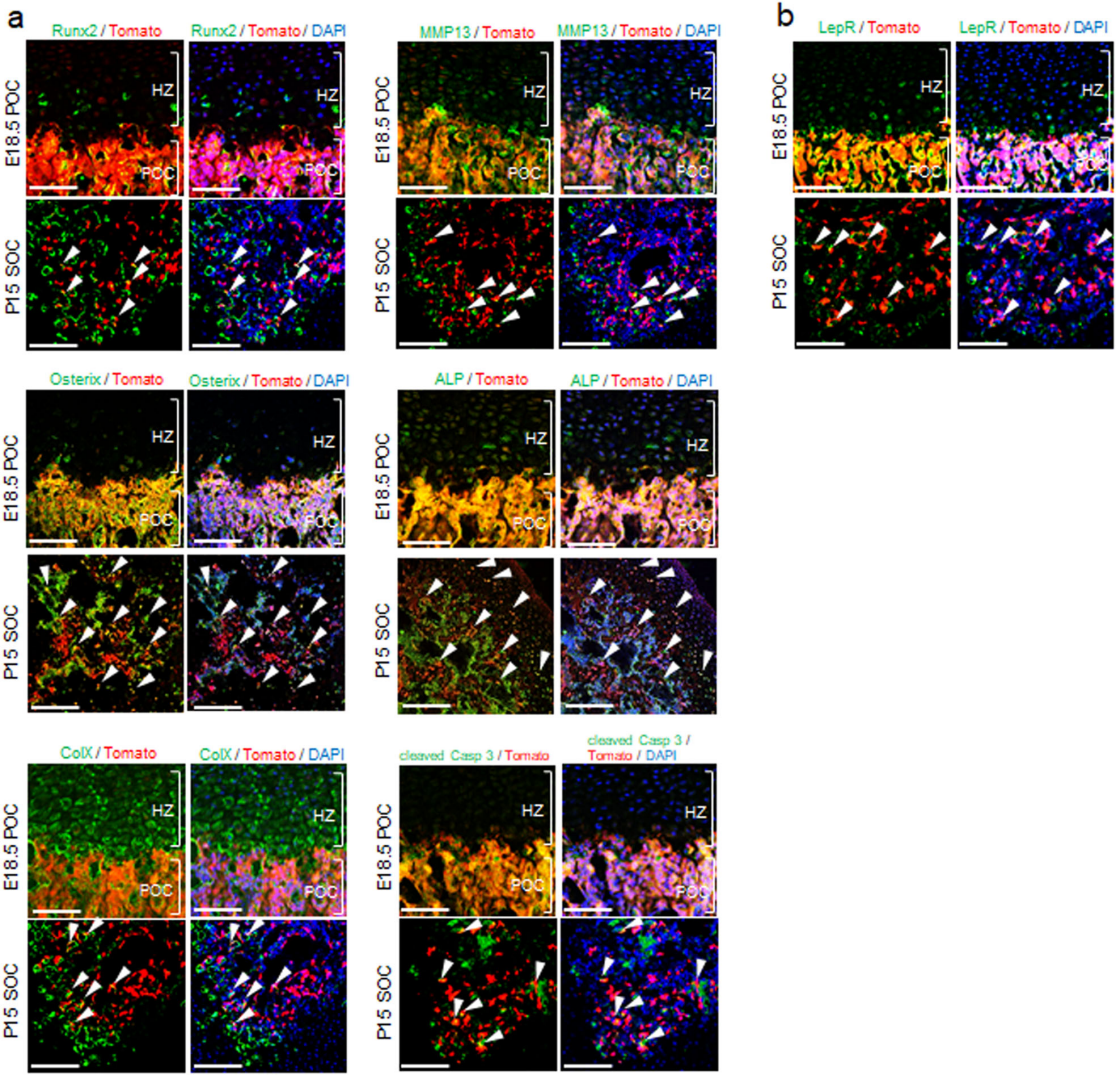
LepR^+^ MSCs contribute to endochondral ossification during embryonic and postnatal growth. **a**, **b** Immunofluorescence shows overlapping of Tomato^+^ and the expression of Runx2, Osterix, collagen X, MMP13, alkaline phosphatase, cleaved caspase 3 and LepR in POC (E18.5) and SOC (P15) of *Lepr-cre; tdTomato* mice. Arrowheads indicate the overlapping of Tomato^+^ and the expression of indicated markers. HZ = Hypertrophic zone; POC = Primary ossification center; SOC = Secondary ossification center; ColX=collagen X; ALP = alkaline phosphatase; cleaved Casp 3=cleaved caspase 3. Scale bar, 100 μm.

Because matrix remodeling during endochondral ossification mainly happens in the ossification center during limb development, we further fate mapped the Tomato^+^ cells in POCs and SOCs by immunostaining with antibodies against hypertrophic markers^24^. Immunofluorescence revealed the expression of the hypertrophic markers Runx2, Osterix, collagen X, MMP13 and alkaline phosphatase in Tomato^+^ cells both in POCs and SOCs (Fig. 2a). Interestingly, these Tomato^+^ cells also expressed the apoptotic marker cleaved caspase 3 (Fig. 2a). In the region of hypertrophic zone, we also observed a population of Tomato^−^ cells that also expressed these hypertrophic markers. These hypertrophic chondrocytes are known to be replaced by yet-unidentified osteoprogenitor cells, accompanying vascular invasion, although they have recently been demonstrated to survive and become part of osteogenic cells, and persist into adulthood^25^. Since chondrocytes progress through a series of maturational changes, including hypertrophy and apoptosis, in the process of endochondral ossification^26^, we believe that LepR^+^ MSCs are the cells replacing these hypertrophic chondrocytes during bone growth in POCs and SOCs. It should be noted that an antibody against the LepR extracellular domain exhibited staining in a pattern very similar to that of Tomato expression within POCs and SOCs in *Lepr-cre; tdTomato* conditional reporter mice (Fig. 2b) and overlapped with Tomato expression around sinusoids and arterioles in the bone marrow (Fig. S1c) as consistent with previous study^21^. These data together suggest the involvement of LepR^+^ MSCs in the endochondral ossification process in the ossification centers.

### Deletion of PP2A in LepR^+^ MSCs inhibits cellular proliferation and hypertrophic differentiation within POCs and SOCs

In silico analysis of the regulatory landscape during the osteogenic differentiation of MSCs^27^ revealed that the most transcriptionally downregulated genes were cell cycle-associated genes (Fig. S2a, b). Among these, the cyclin D1 or E1 and Cdk6 genes exhibited the greatest decreases during MSC osteogenesis (Fig. S2c), and they were associated with several PP2A subunits, as revealed by the Ingenuity Pathway Analysis (IPA) software (Fig. S2d). During endochondral ossification, both collagen X and MMP13 transcription is controlled by Runx2^8,28^, and the activity of Runx2 is controlled by the cell cycle regulator cyclin D1-Cdk4, which phosphorylates Runx2 at serine-472 (S472) and induces Runx2 degradation in a ubiquitination-proteasome-dependent manner^10^. PP2A is a key player in regulating Treg cell function through its serine/threonine protein phosphatase activity^15^; however, its direct role in regulating bone growth during limb development is not known.

**Fig. 3:**
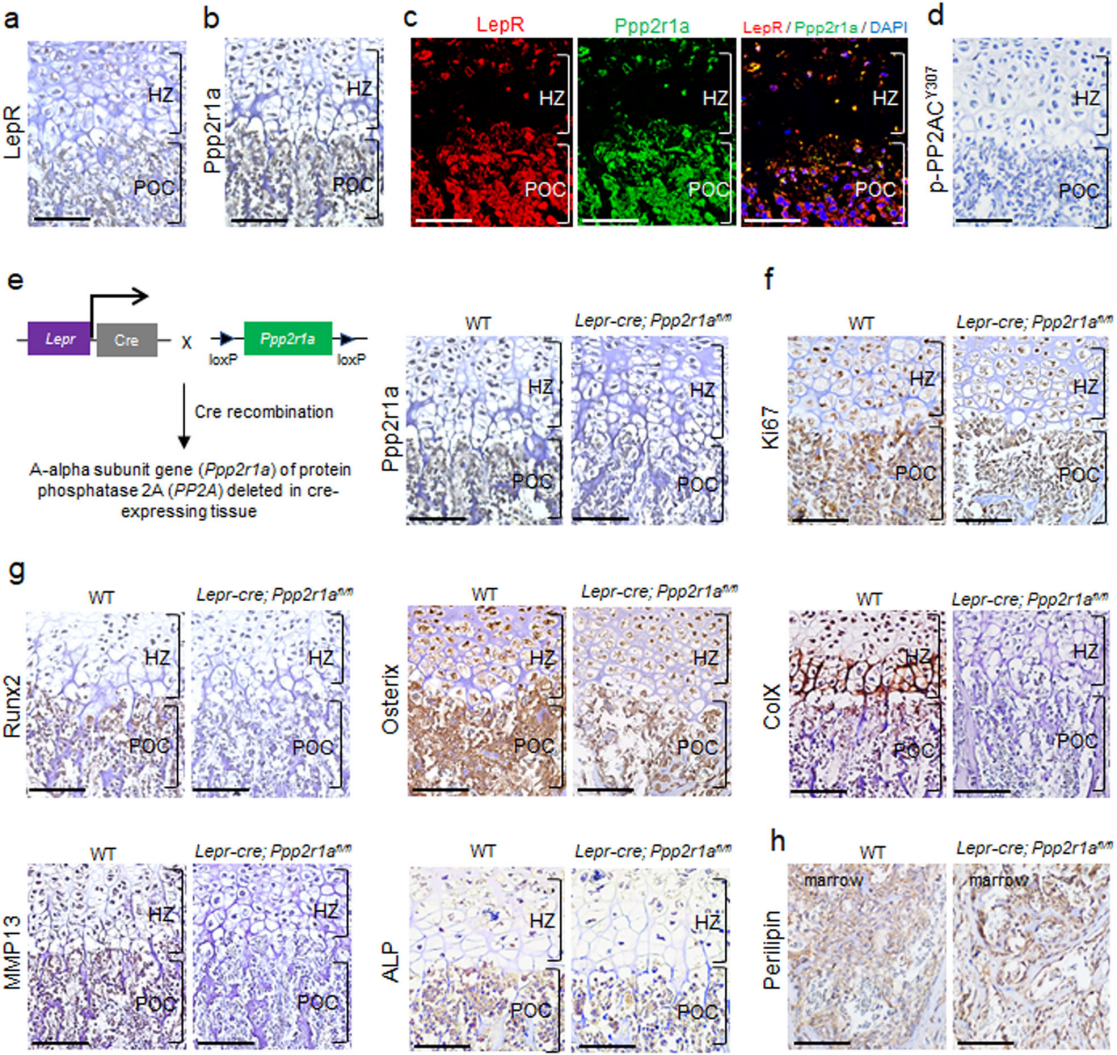
Deletion of PP2A in LepR^+^ MSCs inhibited proliferation and hypertrophic differentiation of POC in embryonic stage. Sections of E18.5 distal femur from *Lepr-cre; Ppp2r1a*^*fl/fl*^ mice were subjected to immunostaining. **a**–**d** Immunohistochemistry (IHC) and immunofluorescence reveal that LepR^+^ MSCs express Ppp2r1a and unphosphorylated (Y307) PP2AC in POC. **a** LepR IHC. **b** Ppp2r1a IHC. **c** Double immunofluorescence of LepR and Ppp2r1a. **d** Unphosphorylated (Y307) PP2AC IHC. **e** Illustration of conditional knockout of *Ppp2r1a* in LepR^+^ MSCs. IHC reveals successful deletion of *Ppp2r1a* in LepR^+^ MSCs in POC. IHC reveals that deletion of *Ppp2r1a* in LepR^+^ MSCs leads to decreased expression of Ki67 (**f**) and hypertrophic markers, such as Runx2, Osterix, collagen X, MMP13 and alkaline phosphatase (**g**), and increased expression of Perilipin (**h**) in POC at E18.5. The rectangle box shows magnified picture. HZ = Hypertrophic zone; POC = Primary ossification center; ColX =collagen X; ALP = alkaline phosphatase. Scale bar, 100 μm.

**Fig. 4:**
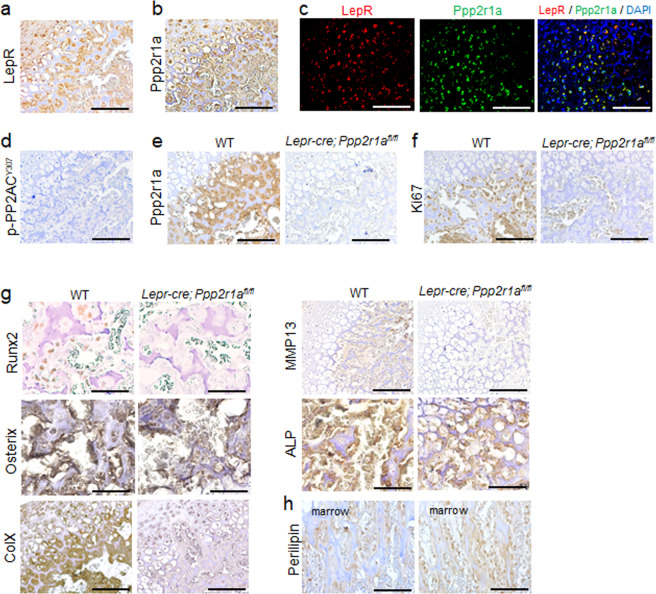
Deletion of PP2A in LepR^+^ MSCs inhibited proliferation and hypertrophic differentiation of SOC in early postnatal stage. Sections of P15 distal femur from *Lepr-cre; Ppp2r1a*^*fl/fl*^ mice were subjected to immunostaining. **a–d** Immunohistochemistry (IHC) and immunofluorescence reveal LepR^+^ MSCs express Ppp2r1a and unphosphorylated (Y307) PP2AC in SOC. (a) LepR IHC. **b** Ppp2r1a IHC. **c** Double immunofluorescence of LepR and Ppp2r1a. **d** Unphosphorylated (Y307) PP2AC IHC. **e** IHC reveals successful deletion of *Ppp2r1a* in LepR^+^ MSCs in SOC. IHC reveals that deletion of *Ppp2r1a* in LepR^+^ MSCs leads to decreased expression of Ki67 (**f**) and hypertrophic markers, such as Runx2, Osterix, collagen X, MMP13 and alkaline phosphatase (**g**), and increased expression of Perilipin (**h**) in SOC at P15. HZ = Hypertrophic zone; POC = Primary ossification center; ColX =collagen X; ALP = alkaline phosphatase. Scale bar, 100 μm.

Immunofluorescence analysis revealed that LepR and Ppp2r1a (a scaffold protein of the PP2A complex) were expressed in almost all cells in E18.5 POCs (Fig. 3a–c). Moreover, these cells did not express phosphorylated PP2AC (Y307) at this stage (Fig. 3d). As the phosphorylation of PP2A catalytic protein at tyrosine 307 renders it inactive, this result suggests that PP2A activity was not suppressed. *Lepr-cre; Ppp2r1a*^*fl/fl*^ mice that had been genetically modified to conditionally delete *Ppp2r1a* did not express Ppp2r1a in POC sections (Fig. 3e), exhibited decreased expression of Ki67 (Fig. 3f), Runx2, Osterix, collagen X, MMP13 and alkaline phosphatase (Fig. 3g), but increased expression of perilipin (the differentiation marker of adipocytes) (Fig. 3h). Similarly, we observed that LepR and Ppp2r1a were also expressed in almost all cells in P15 SOCs (Fig. 4a–c). Moreover, these cells did not express phosphorylated PP2AC (Y307) at this stage (Fig. 4d), suggesting that PP2A was activated. *Lepr-cre; Ppp2r1a*^*fl/fl*^ mice (Fig. 4e) also showed decreased expression of Ki67 (Fig. 4f), Runx2, Osterix, collagen X, MMP13 and alkaline phosphatase (Fig. 4g), but increased expression of perilipin in SOC sections (Fig. 4h). In addition to the suppression of endochondral ossification due to loss of Runx2, Osterix, collagen X, MMP13 and alkaline phosphatase expression, the conditional deletion of *Ppp2r1a* expression also led to a reduction in cell size within POCs at E18.5 (Fig. S3a), but not in SOCs at P15 (Fig. S3b). These data together suggest that the *Lepr-cre; Ppp2r1a*^*fl/fl*^ mice exhibit impaired chondrocyte hypertrophy and cell enlargement at embryonic stages but not at postnatal stages.

### *Lepr-cre; Ppp2r1a*^*fl/fl*^ mice exhibit reduced bone density and increased fat density in trabecular bones

**Fig. 5:**
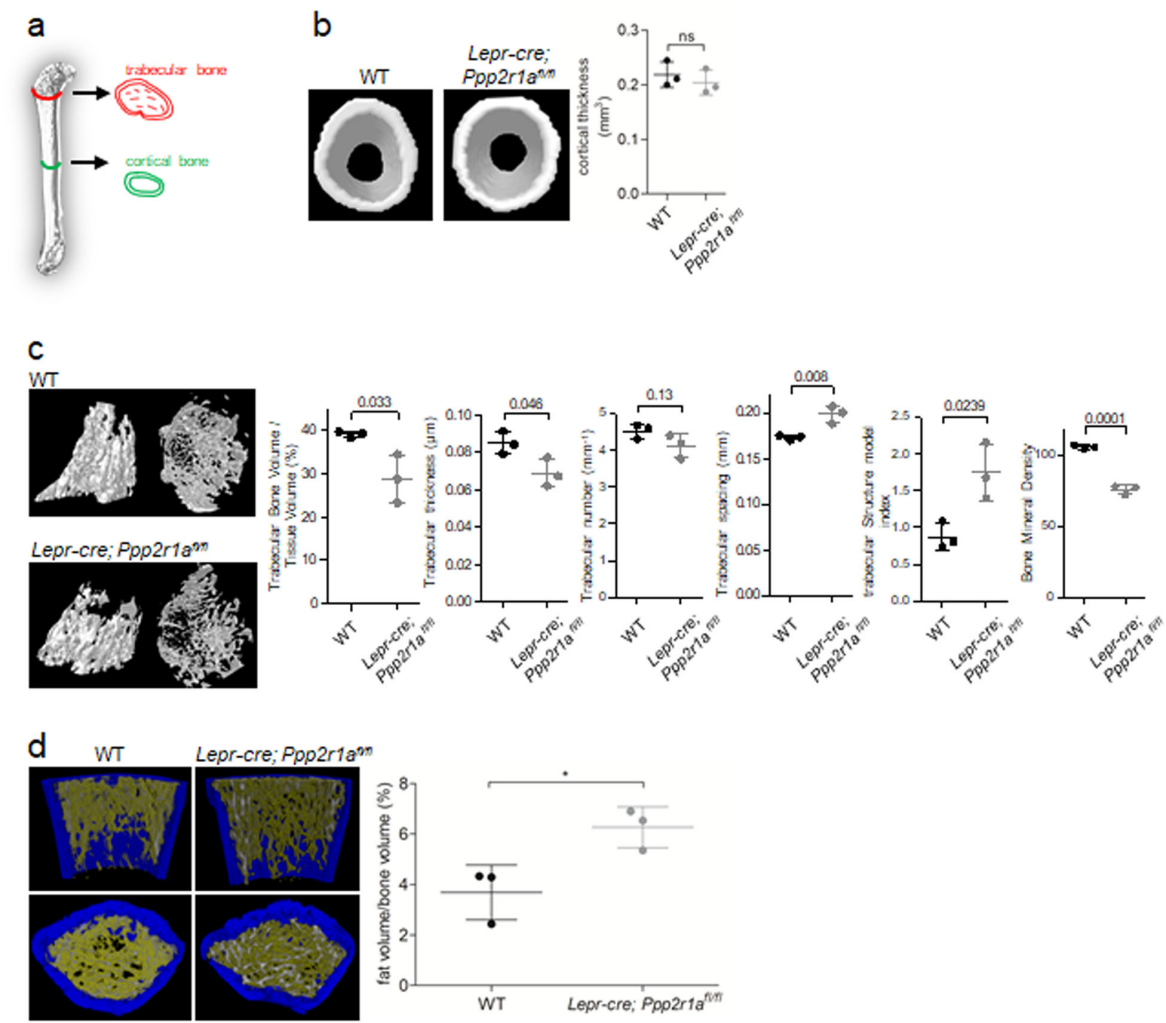
The *Lepr-cre; Ppp2r1a*^*fl/fl*^ mice exhibited reduced bone density and increased fat density in trabecular bones. Micro-CT analysis of femur in *Lepr-cre; Ppp2r1a* ^*fl/fl*^ mice and relative WT littermates at P30. **a** Illustration of areas scanned for cortical and trabecular bone. **b left** Morphological characteristics of femur cortical bone. **b right** There is no significant difference in cortical thickness between *Lepr-cre; Ppp2r1a* ^*fl/fl*^ mice and relative WT littermates. **c left** Morphological characteristics of femur trabecular bone. **c right** Quantification of micro-CT data on trabecular bone volume fraction (Trabecular Bone Volume / Tissue Volume, %), trabecular thickness (μm), trabecular number (mm^−1^), trabecular spacing (mm), trabecular structure model index, and bone mineral density (*p* values represent in number on top of each assessment as determined with Student’s *t*-test). **d left** Trabecular bone architecture in WT and *Lepr-cre; Ppp2r1a* ^*fl/fl*^ mice with labeling using software analysis of μCT sections (blue: cortical bone; white: trabecular bone; yellow: fat). **d right** Comparison of fat volume/bone volume (%) in μCT image analysis of *Lepr-cre; Ppp2r1a* ^*fl/fl*^ and relative WT littermates. *n* = 3 mice; Data are mean ± s.d. *p < 0.05 as determined with Student’s *t*-test. ns, not significant.

To assess the effects of PP2A deletion on osteogenic and adipogenic differentiation of LepR^+^ MSCs, micro-computed tomography (micro-CT) analysis was performed to obtain quantitative measurements of bone and fat densities at P30 (Fig. 5a). At this time point, endochondral ossification in SOC is complete but bone growth in the epiphyseal plate cartilage has not yet started, and therefore, the influence of growth plate expansion on bone content and length is minimal. With regard to the distal femur, PP2A deletion did not induce changes in the cortical thickness of diaphyses (Fig. 5b), whereas it reduced trabecular bone volume to tissue volume ratio, trabecular number, trabecular thickness, and bone mineral density, and increased trabecular spacing and trabecular structure model index relative to wild-type littermate mice (Fig. 5c). Moreover, PP2A deletion increased the fat volume percentage in bone (Fig. 5d). These data together suggest that PP2A deletion suppresses osteogenic differentiation and enhances adipogenic differentiation in LepR^+^ MSCs.

### PP2A deletion in LepR^+^ MSCs inhibits osteogenesis and chondrogenesis but promotes adipogenesis in vitro

**Fig. 6:**
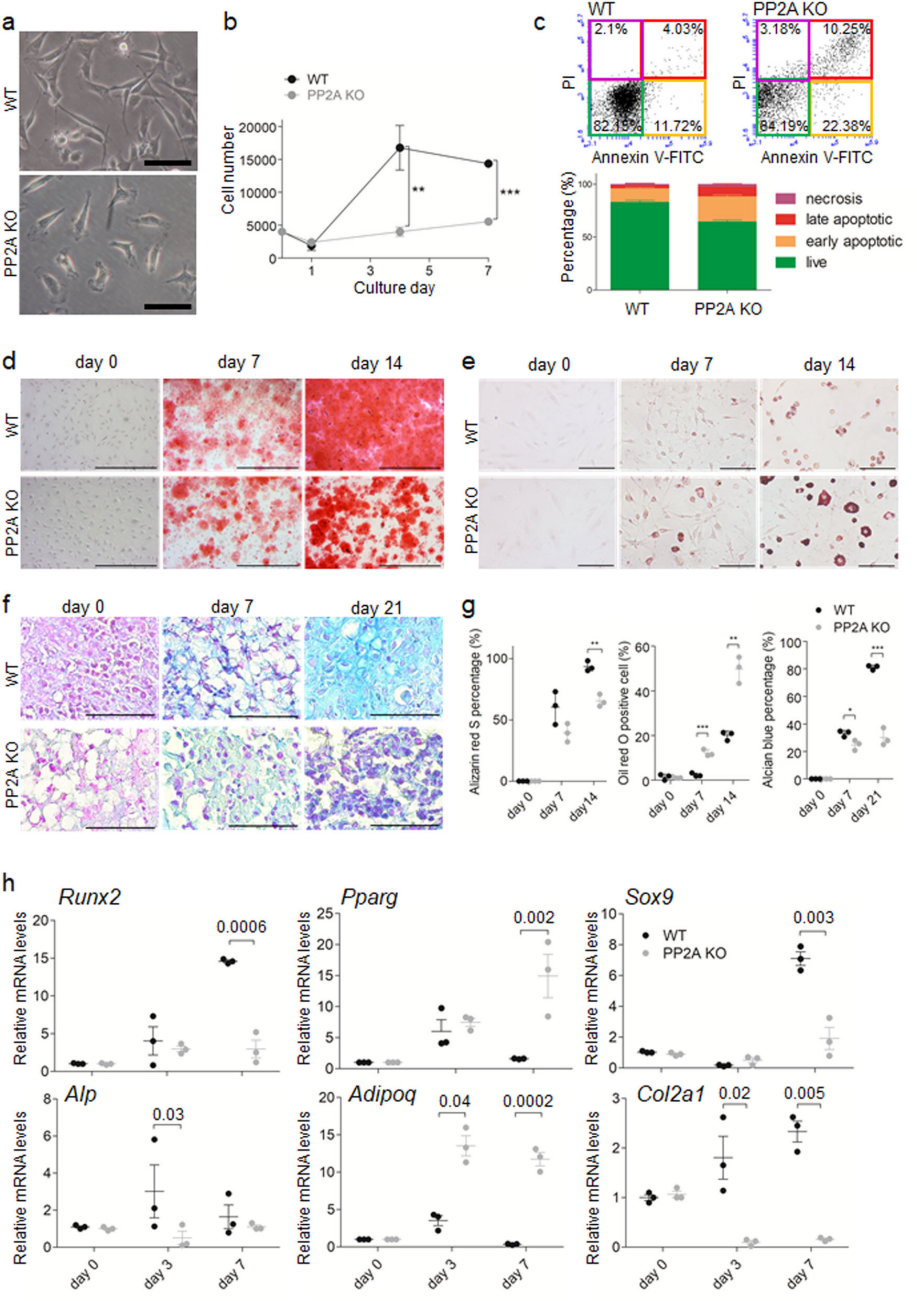
PP2A deletion in LepR^+^ MSCs inhibits osteogenesis and chondrogenesis but promotes adipogenesis in vitro. MSCs isolated from WT (WT MSCs) and *Lepr-cre; Ppp2r1a* ^*fl/fl*^ mice (PP2A KO MSCs) were subjected to analysis. **a** WT and PP2A KO MSCs exhibit no difference in morphology. Scale bar, 50 μm. **b** PP2A KO MSCs exhibit decreased proliferation. *n* = 3 independent experiments. **c** Represented flow cytometry plots of Annexin V-FITC/PI assay for apoptosis analysis of WT and PP2A KO MSCs. After FSC/SSC gating (left), MSCs are categorized into necrosis, late apoptosis, early apoptosis and live stages (right). The corresponding percentage are shown in bar chart. **d** WT and PP2A KO MSCs were induced for osteogenic differentiation and stained with Alizarin Red S at different time points. Scale bar, 1000 μm. **e** WT and PP2A KO MSCs were induced for adipogenic differentiation and stained with Oil red O at different time points. **f** WT and PP2A KO MSCs in micromass were induced for chondrogenic differentiation and stained with Alcian blue & nuclear fast red at different time points. Scale bar in **e**, **f**, 100 μm. **g** Quantification percentage of Alizarin red S staining, Oil red O staining and Alicante blue staining according to different differentiations respectively at each time points. *n* = 3 independent experiments. **h** qPCR analysis of transcript levels for genes associated with osteogenesis (*Runx2*, *Alp*), adipogenesis (*Pparg*, *Adipoq*) and chondrogenesis (*Sox9*, *Col2a1*) differentiation in MSCs induced by differentiation medium at each time points. Transcript levels were normalized based on β-actin amplification. *n* = 3 independent experiments. *p < 0.05; **p < 0.01; ***p < 0.001 as determined with Student’s *t*-test. Data are mean ± s.d.

To determine the molecular mechanism involved in PP2A deletion-induced suppression of osteogenic differentiation and enhancement of adipogenic differentiation, we isolated and expanded PP2A KO MSCs and WT MSCs. These two cell populations did not exhibit differences in morphology (Fig. 6a). However, PP2A KO MSCs exhibited a decrease in proliferation (Fig. 6b) and increased in apoptosis compared to WT MSCs (Fig. 6c). PP2A KO MSCs exhibited a decrease in osteogenic potential (Fig. 6d, g), whereas their adipogenic potential increased (Fig. 6e, g) compared to WT MSCs at different time points. Moreover, we observed that micromass of PP2A KO MSCs decreased in chondrogenic differentiation (Fig. 6f, g) at different time points. The mRNA levels of each differentiation-related genes corresponded to its differentiation result (Fig. 6h). These data suggest that the in vitro phenotypes of PP2A KO MSCs are similar to the limb development phenotypes of *Lepr-cre; Ppp2r1a*^*fl/fl*^ mice, and it may be useful to use the former model to investigate the molecular mechanism mediated by PP2A to affect POCs and SOCs during limb development.

### Runx2 dephosphorylation mediated by PP2A induces chondrocyte hypertrophy

**Fig. 7:**
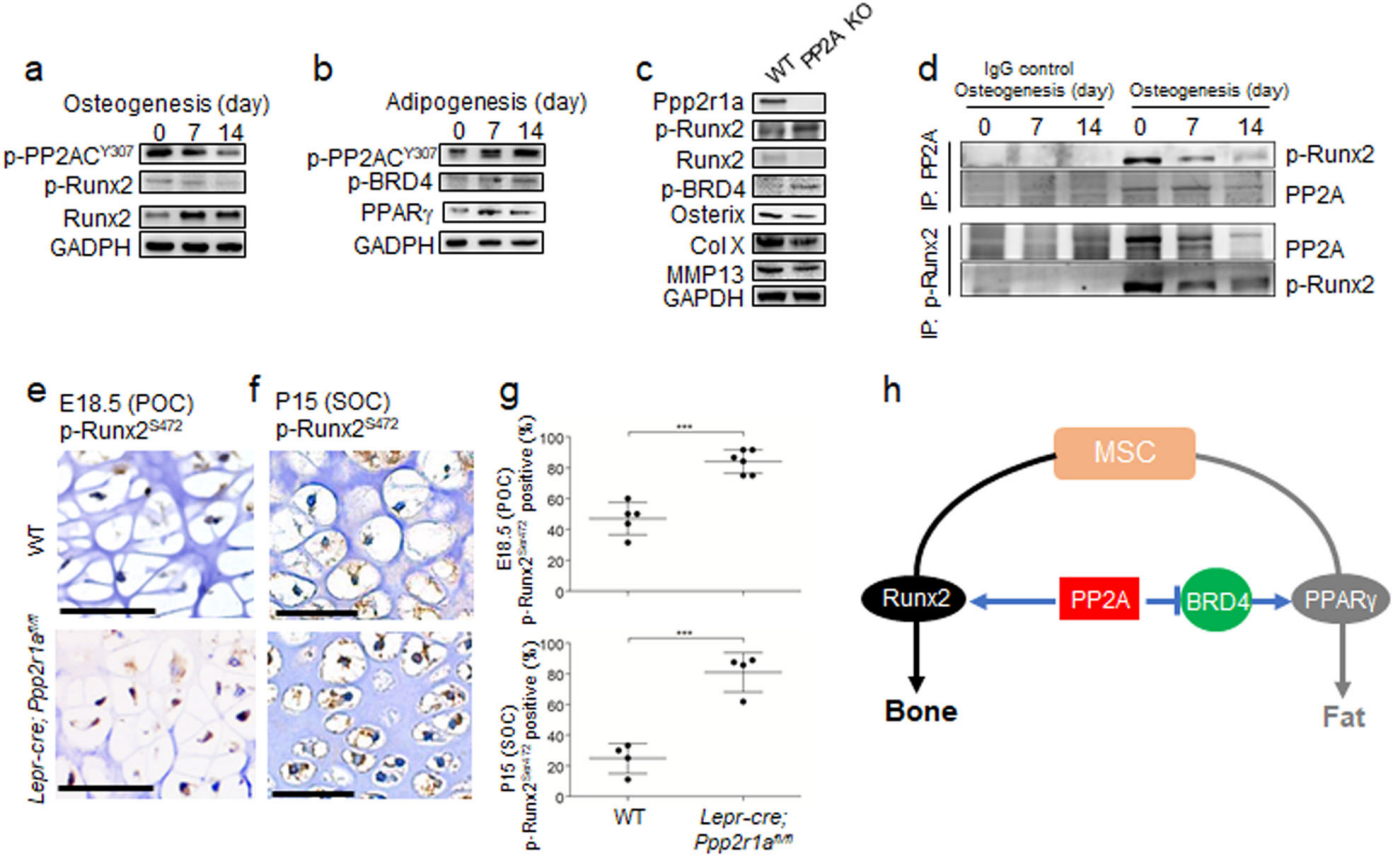
PP2A deletion in LepR^+^ MSCs increases Runx2 phosphorylation at Ser472 and reduces chondrocyte hypertrophy. MSCs isolated from WT (WT MSCs) and *Lepr-cre; Ppp2r1a* ^*fl/fl*^ mice (PP2A KO MSCs) were subjected to analysis. **a** Representative western blot analysis of WT MSCs after osteogenic induction. **b** Representative western blot analysis of WT MSCs after adipogenic induction. **c** Western blot analysis of WT and PP2A KO MSCs. **d** Co-Immunoprecipitation study of cell lysates from WT MSCs with indicated induction for osteogenesis. IP with PP2A-specific or phospho Ser 472 Runx2-specific antibody and precipitates were probed for phospho Ser472 Runx2 and PP2A. **e** and **f** IHC analysis of inactive form phospho Ser472 Runx2 expression in hypertrophic chondrocyte of POC (E18.5) and SOC (P15) from *Lepr-cre; Ppp2r1a* ^*fl/fl*^ mice and relative WT littermate mice. **g** Quantification of phospho Ser472 Runx2 percentage in hypertrophic chondrocyte of POC (E18.5) and SOC (P15) from *Lepr-cre; Ppp2r1a* ^*fl/fl*^ mice and relative WT littermate mice. ***p < 0.001 as determined with Student’s *t*-test. Data are mean ± s.d. **h** Scheme representing regulation of MSC differentiation by PP2A and its substrates protein. Scale bar, 50 μm.

Runx2 is expressed in MSCs during early embryonic development and acts as a master regulator in the commitment of these cells to the osteoblastic lineage^29^. However, Runx2 transcriptional activity is negatively regulated by phosphorylation of its serine residues^30,31^. Moreover, phosphorylation of Runx2 at S472 induces Runx2 ubiquitination and degradation^10^. Interestingly, osteogenic induction reduced the phosphorylation of PP2AC at Y307 and that of Runx2 at S472, as well as increased total Runx2 protein levels in WT MSCs (Fig. 7a). In contrast, adipogenic differentiation increased PP2AC phosphorylation at Y307, BRD4 phosphorylation, which is required for its activity^32^, as well as total Pparγ protein levels in WT MSCs (Fig. 7b). These data highlight the role of PP2A in controlling MSC differentiation by regulating the phosphorylation of Runx2 and the epigenomic reader, BRD4, which co-localizes with lineage-determining transcription factors, such as PPARγ, on active enhancers during adipogenesis^12^. We found increased phosphorylation of Runx2 at S472 and decreased Runx2 protein levels in PP2A KO MSCs compared to WT MSCs (Fig. 7c), which demonstrated the suppression of Runx2 by PP2A deletion. Co-immunoprecipitation assays with antibodies against PP2A and S472-phosphorylated Runx2 further revealed that PP2A was associated with S472-phosphorylated Runx2 in WT MSCs undergoing osteogenic differentiation (Fig. 7d). More importantly, PP2A deletion also increased Runx2 (S472) phosphorylation in E18.5 POCs and P15 SOCs (Fig.7e–g). In addition, we have examined the correlation between phospho-Ser472 Runx2 with Osterix, collagen X, MMP13 and alkaline phosphatase and shows that loss of Ppp2r1a in LepR-MSC increases Runx2 phosphorylation at Ser472 and decreases Osterix, collagen X, MMP13 and alkaline phosphatase expression (Fig. S4) in *Lepr-cre; Ppp2r1a*^*fl/fl*^ mice. These data together suggest that PP2A regulates embryonic and postnatal endochondral ossification through Runx2 phosphorylation.

## Discussion

Previous studies have shown that most bone cells and adipocytes formed in trabeculae and bone marrow are derived from LepR^+^ MSCs; however, these cells only contribute to cortical bone at late stages and are rarely present in articular cartilage and growth plates^21^, while PTHrP^+^ chondrocytes form columnar chondrocytes in the postnatal growth plate, undergo hypertrophy, and become osteoblasts^19^. Since hypertrophic chondrocytes are part of a continuum of the biological process that connects chondrocytes to the osteoblast lineage and leptin plays an important role in the regulation of this process^33^, it is important to understand the contribution of LepR^+^ MSCs to endochondral bone formation. In addition to a previous study that demonstrated that Tomato^+^ cells in *Lepr-cre; tdTomato* mice do not overlap with Col2.3-GFP cells in the cortical bone until the late stages of bone formation (6–14 months postnatally)^21^, we show that Tomato^+^ cells are abundant in POCs and SOCs, but not in the growth plates and articular cartilage. Immunostaining further revealed that LepR^+^ progeny cells were positive for markers of hypertrophic chondrocytes and contribute to endochondral bone formation. Therefore, these studies suggest LepR^+^ MSCs not only contribute to late stage cortical bone formation but also to embryonic and early adult trabecular and bone marrow bone formation through the processes of endochondral ossification.

During the terminal differentiation process of endochondral ossification, growth plate chondrocytes undergo apoptosis with degradation and calcification of cartilaginous matrix followed by deposition of bone matrix. Runx2, a transcription factor that promotes chondrocyte hypertrophy, which regulates transcription of collagen X and MMP13 expressed in hypertrophic zone. Chondrocytes in the center of the prospective bone become hypertrophic and produce collagen X. MMP13, which is produced by hypertrophic chondrocytes and expressed in hypertrophic zone near bone marrow^34^. In the end, the fate of hypertrophic chondrocytes become apoptotic and expressed cleaved caspase 3 (Fig. 2a).

Mechanistically, Pten regulates quiescence, maintenance, and differentiation of LepR^+^ MSCs^21^. LepR^+^ MSCs divide more frequently in *Lepr-cre; Pten*^*fl/fl*^ mice relative to littermate controls. In addition, *Lepr-cre; Pten*^*fl/fl*^ mice have a decreased volume of trabecular and cortical bone but increased adipogenesis in the metaphysis relative to littermate controls. Thus, Pten is required by LepR^+^ MSCs to negatively regulate AKT activation and to maintain normal numbers of quiescent LepR^+^ MSCs in adult bone marrow, as well as to promote osteogenesis and restrain adipogenesis. Interestingly, the expression of Runx2 is also reduced in LepR^+^ MSCs isolated from *Lepr-cre; Pten*^*fl/fl*^ mice relative to littermate controls. However, the mechanism mediated by Pten to suppress Runx2 expression has not yet been studied.

Cyclin D1-Cdk4 phosphorylates Runx2 at S472 and induces Runx2 degradation in a ubiquitination-proteasome-dependent manner^10^. In the current study, we examined the expression and role of PP2A in MSC osteogenesis. We showed that conditional knockout of *Ppp2r1a* in LepR^+^ MSCs reduced Runx2, Osterix, collagen X, MMP13, and ALP expression at POC and SOC, and decreased mRNA levels of *Runx2* and *Alp* and calcium deposition in LepR^+^ MSCs induced for osteogenesis. Besides regulation through Runx2, silencing PP2A Cα in osteoblasts reduced RANKL and increased OPG expression^35^, hence inhibited osteoclast differentiation and activation.

While Yoshida et al.^18^ concluded that reduction of PP2A catalytic subunit alpha promotes in vivo bone formation, their transgenic mice expressed the dominant negative form of PP2A catalytic subunit alpha (substitution of leucine with proline at position 199 in PP2A catalytic subunit alpha), and it is cloned downstream of the 2.3-kb *Col1a1* promoter. The 2.3-kb *Col1a1* promoter was used to study osteoblasts, whereas *Lepr-cre* was used to mark mesenchymal stromal cells. A previous study has also shown that when using *Lepr-cre; tdTomato; Col2.3-GFP* mice, Tomato^+^*Col2.3*-GFP^+^ cells only start to increase at 6 months of age (10%-23% at this stage). These findings suggest that the knockout system is different from that used by Yoshida et al. Furthermore, it is possible that the dominant negative phenotype may in fact be a semi-dominant one. The mammalian PP2A catalytic C subunit has two isoforms (α and β), which are 97% identical, ubiquitously expressed, and highly conserved. Whereas, PP2A Cα and PP2A Cβ seem to be interchangeable in vitro^36^, studies in vivo suggested that both isoforms are not functionally redundant. In mouse model, Ppp2ca (Cα) KO mouse is embryonically lethal with no gastrulation / mesoderm formation and Wnt signaling defected, implying a lack of redundancy between these isoforms. Likewise, total body Ppp2r1a (Aα) KO mice (embryonic lethal in prion-Cre mouse with *loxP*-flanked *Ppp2r1a* gene in all tissue^37^) are neither viable, despite 86% of sequence identity between Aα and Aβ proteins^38^. For this reason, we used conditional KO mice for PP2A A subunit in *Lepr-cre* cells.

Notably, there was no significant difference in cortical bone content between *Lepr-cre; Ppp2r1a*^*fl/fl*^ mice and relative littermate controls at P30. This could be explained by previous observations that LepR^+^ MSCs do not contribute to early cortical bone formation, since Tomato^+^ cells do not overlap with Col2.3-GFP cells at this stage^21^. The effect of conditional PP2A deletion on cortical thickness and mineral content should be further evaluated at the late stages of limb development. Nevertheless, our data support the idea that cortical and trabecular bones should be considered as having different mechanisms that manipulate bone remodeling and the same treatment will result in different effects, although these underlying mechanisms are currently unknown^39^. In contrast to osteogenesis, PP2A deletion led to increased adipogenic potential in LepR^+^ MSCs and increased lipid content in trabecular bone at P30. PP2A, the principal BRD4 serine phosphatase, has been known to associate with and dephosphorylate BRD4, thereby inhibiting its activity^32^. BRD4 binds to active enhancers of adipogenesis, such as PPARγ, to control the expression of cell identity genes and induce adipogenic differentiation^12^. In the present study, induction of adipogenic differentiation in LepR^+^ MSCs was associated with increased phosphorylation of PP2A at Y307 and decreased PP2A activity. As expected, adipogenic differentiation was also associated with increases in the phosphorylation of BRD4 and total protein levels of PPARγ. Collectively, these data suggest that during embryonic and postnatal bone development, PP2A dephosphorylates Runx2 and BRD4, and thus plays a major role in positively and negatively regulating osteogenesis and adipogenesis, respectively (Fig. 7h). Moreover, PP2A Cα reduction in MSC cell line stimulated adipogenesis through attenuating Wnt10b expression and the phosphorylation of GSK-3β^40^. This also led to reduction in total and nuclear β-catenin expression. In current study, we showed that conditional knockout of *Ppp2r1a* in LepR^+^ MSCs increased perilipin expression at both POC and SOC, and increased mRNA levels of *Pparg* and *Adipoq* and fat deposition in LepR^+^ MSCs induced for adipogenesis.There are limitations to the current study that warrant discussion. For example, it has not been shown that which PP2A B subunit isoforms involved in regulating Runx2 dephosphorylation at Ser472. However, B56α expression is increased through adipogenic differentiation and decreased through osteogenic differentiation^41^. In contrast, PR130 expression is reduced through adipogenic and osteogenic differentiation^41^. These studies suggest that several PP2A B subunit isoforms, such as B56α and PR130, are involved in osteogenic and adipogenic differentiations.

In conclusion, the current data identified LepR^+^ MSCs as the cell origin of endochondral ossification during embryonic and postnatal bone formation, and that the PP2A-Runx2 and PP2A-BRD4 pathways play primary roles in regulating the differentiation potential of MSCs. Since endochondral ossification is involved in normal bone and cartilage remodeling, such as fracture healing and several pathological bone diseases, these data also suggest that PP2A may be a novel therapeutic target in the treatment of dysregulated bone formation.

## Methods

### Mice

*Ppp2r1a*^*flox/flox*^ mice, carrying conditional alleles with loxP sites flanking exon 5–6 of *Ppp2r1a* or *Rosa26-CAG-loxp-stop-loxp-tdTomato* mice were crossed to *Lepr-cre* mice^42^ and maintained in C57BL/6 background. Mice were purchased from the Jackson Laboratory. Postnatal and adult bone were obtained from male mice, while embryonic mice were obtained from either sex. All animal studies and care of live animals were approved and performed following the guidelines made by the China Medical University Institutional Animal Care and Use Committee 2018-040.

### Bone sectioning and immunostaining

*Lepr-cre; tdTomato* samples were prepared and immunostained according to methods described before^43^. In brief, 25 μm sections were stained overnight at 4 °C with anti-LepR (Proteintech 20966-1-AP, 1:10), anti-Ki67 (Millipore AB9260, 1:100), anti-Osterix (Bioss bs-1110, 1:50), anti-Runx2 (Cell Signaling 8486, 1:50), anti-collagen X (Abcam ab58632, 1:50), anti-MMP13 (Genetex GTX-100665, 1:100), anti-alkaline phosphatase (Genetex GTX-100817, 1:100) and anti-cleaved caspase 3 (Cell Signaling 9661, 1:400). The secondary antibodies used were DyLight^®^ 650-conjugated goat anti-rabbit (Bethyl A120-101D5, 1:50) and goat anti-rabbit IgG antibody-FITC (Bethyl A120-201F, 1:50). Nuclear counterstaining was achieved by the use of mounting with anti-fade medium with DAPI. Images were acquired with Leica SP2 / SP8X confocal spectral microscope in Medical Research Core Facilities, Office of Research & Development in China Medical University.

For immunohistochemistry (IHC), dissected bones were fixed in 4% paraformaldehyde overnight followed by 1-day decalcification in 0.5 M EDTA. Bones were embedded in paraffin and sectioned into 10 μm thickness. Sections were first subjected to antigen retrieval and permeabilized by 0.3% (vol/vol) Triton X-100 in PBS for 15 min and endogenous peroxidase activity was blocked with 3% hydrogen peroxide. Sections were then immersed in PBS with 10% BSA for 30 min and then stained overnight at 4 °C with anti-LepR, anti-Ppp2r1a (Genetex GTX-102206, 1:100), anti-phospho Tyr307 PP2AC (Santa Cruz sc-271903, 1:50), anti-Ki67, anti-Osterix, anti-Runx2, anti-collagen X, anti-MMP13, anti-alkaline phosphatase, anti-Perilipin (Millipore ABS526, 1:100) and anti-human phospho Ser451 Runx2 (corresponding to Ser472 in the murine Runx2, Bioss bs-5685, 1:50). HRP-conjugated secondary antibody was then added for 30 min and the detections were done by DAB substrate. Sections were then washed in PBS and nuclei counterstained with Gill’s hematoxylin. For double immunofluorescence, fluorescent-dye conjugated secondary antibodies was added for 30 min and nuclear counterstaining was achieved by the use of DAPI.

### Cell culture

Intact femurs and humeri were dissected carefully from the mice body with the muscle and connective tissue removed from the bones. The bones were cut at both ends at the site near to the marrow cavity. A 26-gauge needle was inserted at the exposed end of the bones and the marrow plug was flushed out by 1% BSA in PBS. Cell suspension was collected through a 70-mm filter mesh to remove any bone spicules or muscle and cell clumps. MSCs were isolated and expanded with protocols described previously^44^. In brief, bone marrow cells were cultured in 100-mm culture dishes with complete alpha MEM medium (with 16.6% FBS) and incubated at 37 °C with 5% CO_2_ in a humidified chamber. After 12 h, the nonadherent cells were removed on the surface of the dish by changing the medium. Thereafter, this step was repeated every 24 h for up to 72 h of initial culture. Within 4–8 days, the culture becomes more confluent and distinct fibroblastic cells reaches 65–70% confluence within 2 weeks. After 2 weeks of initiating culture, the culture was trypsinized with 0.05% trypsin/1 mM ethylenediaminetetraacetic acid for 2 min at 37 °C. The trypsin was neutralized by adding 5 ml of complete medium, and culture in a 100-mm dish. The culture medium was changed every 3 days. Typically, cell confluence is achieved in 7 days.

### Flow cytometry and Annexin V-PI analysis

For detecting apoptosis percentage, MSCs were resuspended in 10^6^ cells/tube and incubated by Annexin V-FITC and Propidium Iodide at RT for 15 minutes in dark according to the manufacturer’s instructions (Strong Biotech Corporation AVK050). Analysis was taken by BD Accuri™ C6 Plus Flow Cytometer.

### In vitro differentiation

Osteogenic differentiation was induced with 10^−8^M dexamethasone (Sigma D1756), 50 μg/ml ascorbic acid (Sigma A4406) and 10^−2^M β-glycerophosphate (Fluka 50020) for 7 and 14 days in alpha MEM with 10% FBS. Adipogenic differentiation was induced with alpha MEM (with 10% FBS) consisted of 0.05 mM indomethacin (Sigma I7378), 10 μg/ml insulin (Sigma I0516), 10^−8^M dexamethasone, 50 μg/ml ascorbic acid and 0.45 mM 3-isobutyl-1-methylxanthine (IBMX, Sigma I5879) for 7 or 14 days. For chondrogenic differentiation, 2.5 ×10^5^ cells/ml was transferred into 15-ml tube (Orange Scientific 5540300) and spin down. After 24 h, the cell pellet will form the ball-like micromass and transfer the medium into DMEM (Hyclone SH30081) with 10^−5^M dexamethasone, 1% NEAA (Sigma M7145), 0.1% ITS + (Sigma I2521), 50 μg/ml ascorbic acid, 40 μg/ml l-proline (Sigma P5607), 100 μg/ml sodium pyruvate (Sigma P5280) and 10 ng/ml TGF-β1 (Sigma T7039) contained. The micromass was cultured by shaking every other day to prevent adherence to the tube and culture for 7 or 21 days. Osteogenic, adipogenic differentiation and chondrogenic micromass sections were detected by staining with Alizarin red S (Sigma A5533), Oil red O (Sigma O9755) and Alcian blue (Sigma B8438) / nuclear fast red (Sigma N3020), respectively.

### Quantitative reverse-transcription PCR (qRT-PCR)

WT and PP2A KO MSCs were differentiated by 0, 3 and 7 days. Total RNA of each differentiated cells was extracted by PureLink™ RNA Mini Kit according to the manufacturer’s instructions (Invitrogen). Total RNA was subjected to reverse transcription and then qRT-PCR using SYBR green on CFX96 Optical Reaction Module (Bio-Rad). Primers used in this study were: β-actin: 5′-

ACTATTGGCAACGAGCGGTT-3′ and 5′-ACACTTCATGATGGAATTGAATGTAGT-3′; *Runx2*: 5′-TTACCTACACCCCGCCAGTC-3′ and 5′-TGCTGGTCTGGAAGGGTCC-3′; *Alp*: 5′-TGAGCGACACGGACAAGA-3′ and 5′-GGCCTGGTAGTTGTTGTGAG-3′; *Pparg*: 5′-GGATAAAGCATCAGGCTTCC-3′ and 5′-TCAATCGGATGGTTCTTCG-3′; *Adipoq*: 5′-GGCCACTTTCTCCTCATTTC-3′ and 5′-AACAGGAGAGCTTGCAACAG-3′; *Sox9*: 5′-AGAACAAGCCACACGTCAAG-3′ and 5′-CAGCAGCCTCCAGAGCTT-3′; *Col2a1*: 5′-TATGGAAGCCCTCATCTTGC-3′ and 5′-CAGATAATGTCATCGCAGAGG-3′.

### Micro-CT

Intact bones were dissected and fixed in 10% neutral buffered formalin for 72 h and were scanned at an isotropic voxel size of 4 μm (80 kV, 450 μA and 2000 ms integration time) at National Laboratory Animal Center, Taiwan by high-resolution X-ray microtomography SkyScan 1076 imaging system (SkyScan, Kontich, Belgium).

### Western blot and immunoprecipitation

Cells were collected by centrifugation, washed in PBS, and lysed in RIPA buffer containing protease and phosphatase inhibitors (Thermo 78442) with vortex vigorously. Lysates were then centrifuged at 11,000*g* at 4 °C for 10 min and the supernatant (quantify the concentration first) was transferred into 4× diluted Laemmli sample buffer containing 2-mercaptoethanol and boiled in 100 °C for 5 min. After that, lysates were separated on 10% Bis-Tris polyacrylamide gels and transferred to PVDF membrane (Millipore IPVH85R). The blots were incubated with primary antibodies overnight at 4 °C and then with secondary antibodies. Blots were developed with the Western Lightening® Plus ECL kit (PerkinElmer). The primary antibodies including anti-Ppp2r1a (Genetex GTX-102206, 1:500), anti-phospho Tyr307 PP2AC (Santa Cruz sc-271903, 1:100), anti-Runx2 (Cell Signaling 8486, 1:1000), anti-human phospho Ser451 Runx2 (Bioss bs-5685, 1:300), anti-PPARγ (ABclonal A0270, 1:500), anti-phospho Ser492 BRD4 (Millipore ABE1451, 1:500), anti-Osterix (Bioss bs-1110, 1:300), anti-collagen X (Abcam ab58632, 1:100), anti-MMP13 (Genetex GTX-100665, 1:500) and anti-GAPDH (Genetex GTX-100118, 1:5000) were used. Immunoprecipitation was performed by Capturem™ IP & Co-IP Kit (Takara, 635721) and the results were analyzed with blotting the elution by anti- Ppp2r1a and anti- human phospho Ser451 Runx2.

### Functional annotation

RNA sequencing raw data from Håkelien et al.^27^ were recompiled. Transcripts were compared between osteogenic differentiated (28 day) to non-differentiated (0 day). Enrichment analysis using Gene Ontology (GO) enrichment analysis to generate cellular and molecular function of the transcripts that are significantly different and using STRING database (http://string-db.org/) to construct the interaction network of cell cycle proteins with PP2A under osteogenic differentiation. Kyoto Encyclopedia of Genes and Genomes (KEGG) enrichment analysis was used to present gene expression in cell cycle.

### Statistics and reproducibility

Multiple group comparisons were analyzed using Analysis of variance (ANOVA) (Tukey) and two group comparisons were analyzed using the two-tailed Student’s *t*-test from GraphPad Prism ver. 5 software. A *p*-value less than 0.05 was considered significant.

### Reporting summary

Further information on research design is available in the Nature Research Reporting Summary linked to this article.

## Supplementary information

Supplementary Information

Description of Supplementary Files

Supplementary Data 1

Reporting Summary

## Data Availability

The authors declare that the data supporting the findings of this study are available within the paper and its Supplementary Information files. Source data for the figures are available in Supplementary Data 1.
