# Supplementary material for: PP2A in LepR+ mesenchymal stem cells contributes to embryonic and postnatal endochondral ossification through Runx2 dephosphorylation
Source: Commun Biol. 2021 Jun 2;4:658. doi: 10.1038/s42003-021-02175-1 (PMC8172534; doi:10.1038/s42003-021-02175-1)
Supplement: Supplementary file 1 — Supplementary Information [file 42003_2021_2175_MOESM1_ESM.pdf]

Supplementary Figure S1. Fate mapping of LepR$^+$ MSCs in POC and SOC during embryonic and postnatal growth which are abundant around sinusoids throughout the bone marrow.

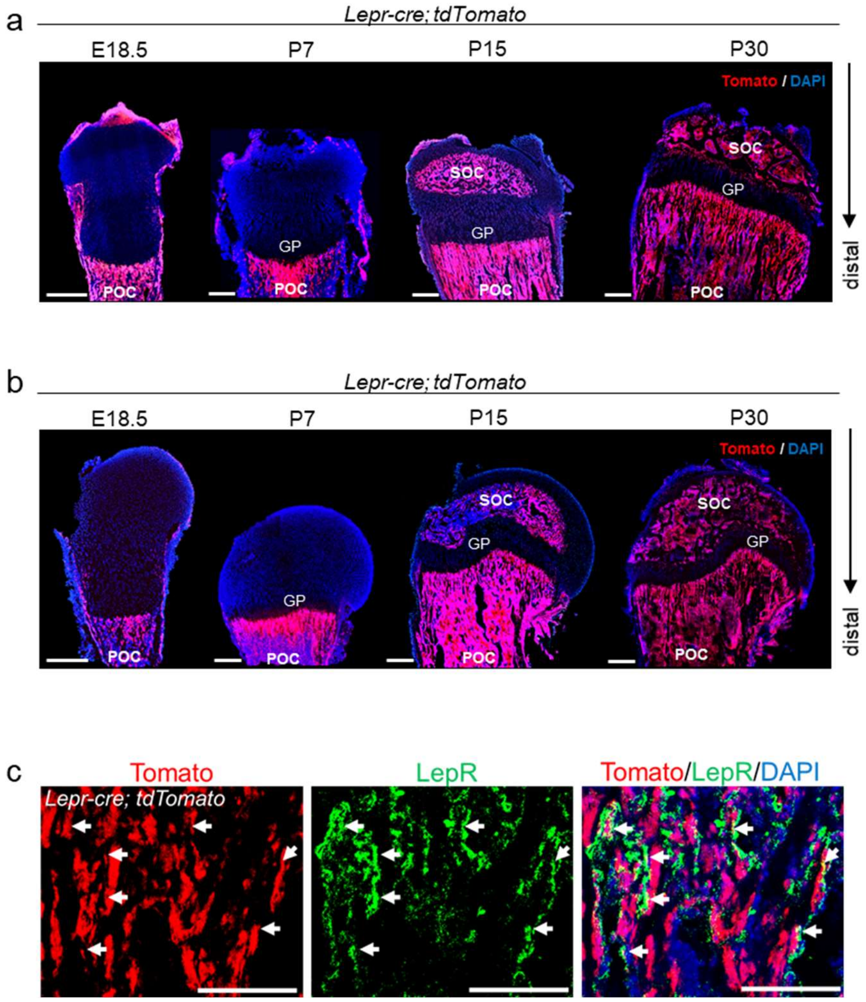

The POC and SOC in **a** the proximal tibia and **b** proximal humerus of *Lepr-cre; tdTomato* mice were histologically examined on E18.5, P7, P15 and P30. Representative pictures showing the appearance of Tomato$^+$ cells at indicated stages. Tomato$^+$ (red) and DAPI (blue). GP=Growth plate; POC=Primary ossification center; SOC=Secondary ossification center. Scale bar, 300 μm. **c** Staining with anti-LepR antibody overlapped with Tomato expression around sinusoids and arterioles in the bone marrow of *Lepr-cre; tdTomato* mice. Arrows depict where Tomato$^+$ overlapped with LepR expression. Scale bar, 100 μm.

Supplementary Figure S2. Gene ontology analysis reveals gene upregulation and downregulation in human MSC undergoing osteogenic differentiation.

a

	Description	*p*-value	*q*-value
Up	cotranslational protein targeting to membrane	3.57E-24	2.01E-20
	SRP-dependent cotranslational protein targeting to membrane	7.69E-24	2.16E-20
	embryonic skeletal system morphogenesis	1.13E-05	0.001292
	bone mineralization	4.18E-05	0.00356
	cell aging	8.98E-05	0.006232
	epithelial cell apoptotic process	0.000714	0.030868
	chondrocyte differentiation	0.002216	0.061357
	post-Golgi vesicle-mediated transport	0.003839	0.086988
	response to vitamin	0.004436	0.094426

	Description	*p*-value	*q*-value
Down	Cell cycle	3.56E-13	1.13E-10
	Focal adhesion	1.47E-10	2.33E-08
	Protein processing in endoplasmic reticulum	5.13E-10	5.42E-08
	Cellular senescence	2.86E-09	2.27E-07
	Oocyte meiosis	1.18E-08	7.48E-07
	Regulation of actin cytoskeleton	2.44E-08	1.29E-06
	Salmonella infection	2.99E-08	1.35E-06
	DNA replication	3.89E-08	1.54E-06
	Proteoglycans in cancer	1.49E-07	4.73E-06

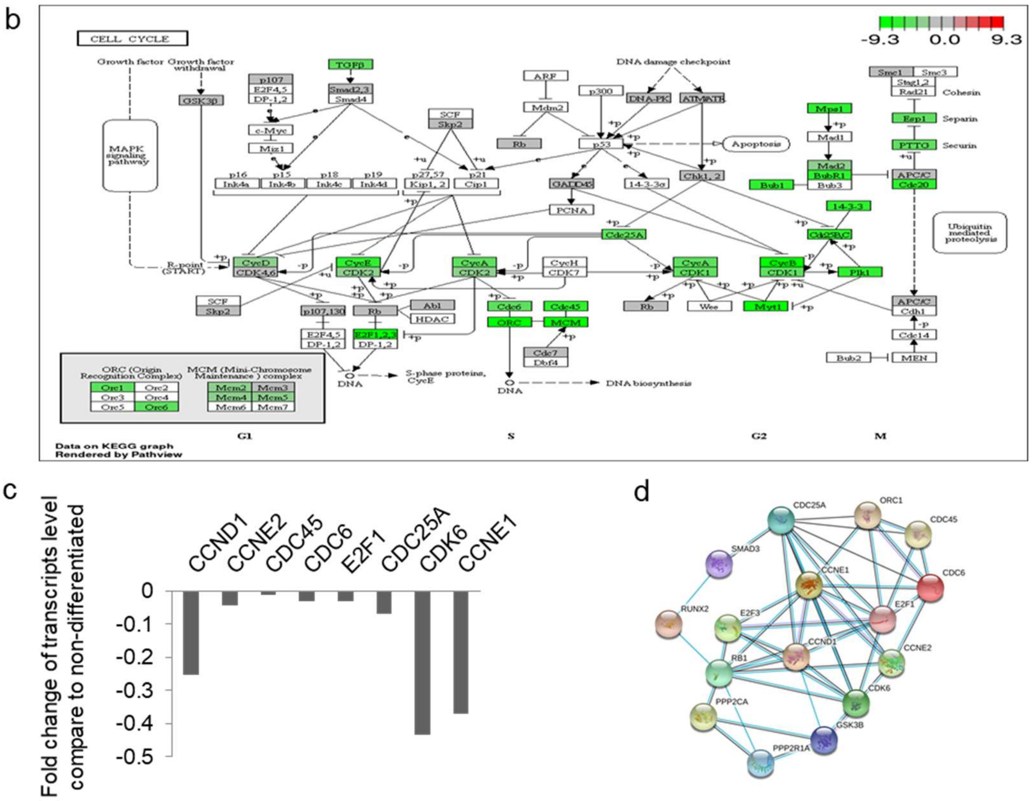

Functional analysis of immortalized MSCs before (day 0) and after osteogenic differentiation for 28 days. **a** 26506 genes analyzed by Gene Ontology (GO) enrichment analysis and categorized by molecular and cellular functions. Upregulated and downregulated gene categories that have false discovery rate (FDR) smaller than 0.05 are shown. The relative *p*-value and *q*-value are indicated. **b** Enrichment map of cell cycle pathway (built by using KEGG hsa04110) with the downregulated molecules shown as green highlights after 28 days of osteogenic differentiation. **c** Downregulated genes associated with cell cycle were filtered by GO enrichment analysis and their transcript levels were compared. The data are shown as the ratios of differentiated (day 28)/un-differentiated (day 0). **d** Relationship of cell cycle proteins with Runx2 and its protein phosphatase PP2A delineated by STRING. RNA-seq data are from Håkelien et al.

Supplementary Figure S3. The *Lepr-cre; Ppp2r1a$^{fl/fl}$* mice exhibited impaired chondrocyte hypertrophy and elongation during embryonic and early postnatal growth.

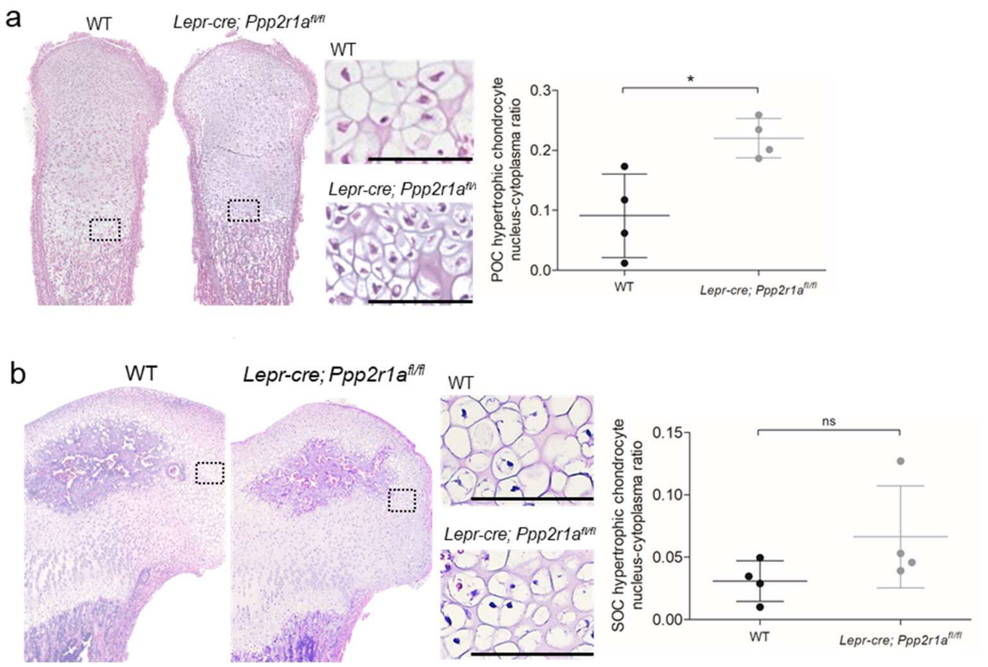

Histological examination of hypertrophic chondrocytes in POC (E18.5) and SOC (P15) of control and *Lepr-cre; Ppp2r1a$^{fl/fl}$* mice. **a** *Lepr-cre; Ppp2r1a$^{fl/fl}$* mice exhibit a significant higher nucleus-cytoplasmic ratio (smaller cell size) in hypertrophic chondrocytes at E18.5 POC. *n* = 4 mice. **b** *Lepr-cre; Ppp2r1a$^{fl/fl}$* mice exhibit a higher nucleus-cytoplasmic ratio (smaller cell size) in hypertrophic chondrocytes at P15 SOC. *n* = 4 mice. *, $p < 0.05$ as determined with Student's *t*-test. ns, not significant. Data are mean ± s.d. Scale bar, 100 μm.

Supplementary Figure S4. PP2A deletion in LepR^{+} MSCs increases Runx2 phosphorylation at Ser472 and decreases osteogenesis and chondrogenesis.

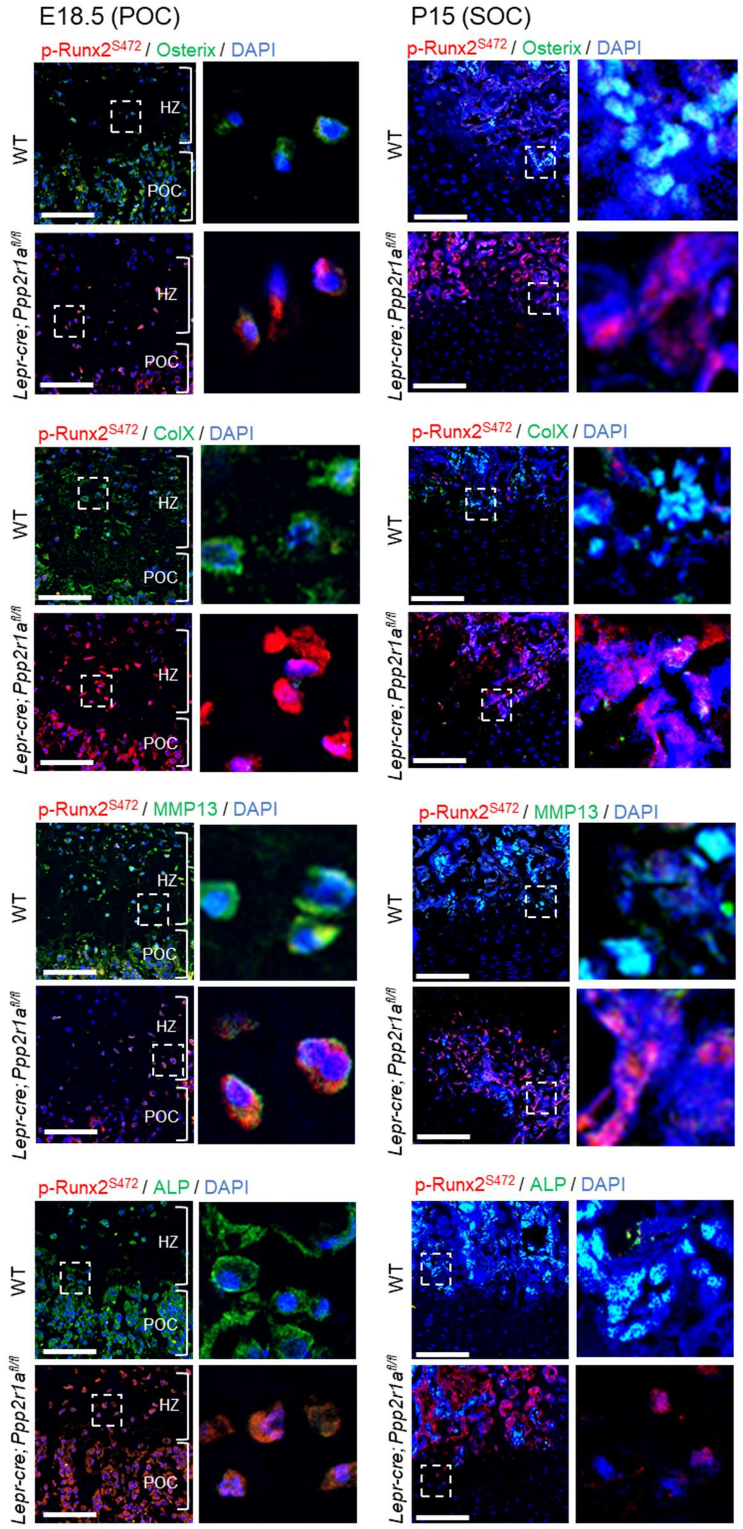

Immunofluorescence shows increased expression of Runx2 phosphorylation at Ser472 and decreased expression of Osterix, collagen X, MMP13 and alkaline phosphatase in *Lepr-cre; Ppp2r1a$^{fl/fl}$* mice in POC (E18.5) and SOC (P15). Magnified images of dash

framed are shown at right panels. HZ=Hypertrophic zone; POC=Primary ossification center; SOC=Secondary ossification center; ColX=collagen X; ALP=alkaline phosphatase. Scale bar, 100 μm.
