# Supplementary material for: PP2A in LepR+ mesenchymal stem cells contributes to embryonic and postnatal endochondral ossification through Runx2 dephosphorylation
Source: Commun Biol. 2021 Jun 2;4:658. doi: 10.1038/s42003-021-02175-1 (PMC8172534; doi:10.1038/s42003-021-02175-1)
Supplement: Supplementary file 2 — Description of Supplementary Files [file 42003_2021_2175_MOESM2_ESM.pdf]

Description of Additional Supplementary Files

File name: Supplementary Data 1

Description: Source data behind the graphs in the paper.
