# Supplementary material for: PP2A in LepR+ mesenchymal stem cells contributes to embryonic and postnatal endochondral ossification through Runx2 dephosphorylation
Source: Commun Biol. 2021 Jun 2;4:658. doi: 10.1038/s42003-021-02175-1 (PMC8172534; doi:10.1038/s42003-021-02175-1)
Supplement: Supplementary file 4 — Reporting Summary [file 42003_2021_2175_MOESM4_ESM.pdf]

nature research

Corresponding author(s): Shih-Chieh Hung

Last updated by author(s): Apr 9, 2021

Reporting Summary

Nature Research wishes to improve the reproducibility of the work that we publish. This form provides structure for consistency and transparency in reporting. For further information on Nature Research policies, see our Editorial Policies and the Editorial Policy Checklist.

Statistics

For all statistical analyses, confirm that the following items are present in the figure legend, table legend, main text, or Methods section.

n/a	Confirmed	
[ ]	[x]	The exact sample size (n) for each experimental group/condition, given as a discrete number and unit of measurement
[ ]	[x]	A statement on whether measurements were taken from distinct samples or whether the same sample was measured repeatedly
[ ]	[x]	The statistical test(s) used AND whether they are one- or two-sided<br>*Only common tests should be described solely by name; describe more complex techniques in the Methods section.*
[x]	[ ]	A description of all covariates tested
[ ]	[x]	A description of any assumptions or corrections, such as tests of normality and adjustment for multiple comparisons
[x]	[ ]	A full description of the statistical parameters including central tendency (e.g. means) or other basic estimates (e.g. regression coefficient) AND variation (e.g. standard deviation) or associated estimates of uncertainty (e.g. confidence intervals)
[x]	[ ]	For null hypothesis testing, the test statistic (e.g. F, t, r) with confidence intervals, effect sizes, degrees of freedom and P value noted<br>*Give P values as exact values whenever suitable.*
[x]	[ ]	For Bayesian analysis, information on the choice of priors and Markov chain Monte Carlo settings
[x]	[ ]	For hierarchical and complex designs, identification of the appropriate level for tests and full reporting of outcomes
[x]	[ ]	Estimates of effect sizes (e.g. Cohen's d, Pearson's r), indicating how they were calculated

Our web collection on statistics for biologists contains articles on many of the points above.

Software and code

Policy information about availability of computer code

Data collection	Immunofluorescence image data were collected using Leica SP2 / SP8X confocal spectral microscope and ANDOR Dragonfly High Speed confocal system. Micro CT data were collected by SkyScan 1076 imaging system (Bruker). Flow cytometry data were collected by BD Accuri C6 Plus.
Data analysis	Immunofluorescence data were analyzed using Leica Application Suite X, Imaris (Bitplane), Image J (NIH) and GraphPad Prism 5.0.

For manuscripts utilizing custom algorithms or software that are central to the research but not yet described in published literature, software must be made available to editors and reviewers. We strongly encourage code deposition in a community repository (e.g. GitHub). See the Nature Research guidelines for submitting code & software for further information.

Data

Policy information about availability of data

All manuscripts must include a data availability statement. This statement should provide the following information, where applicable:

- Accession codes, unique identifiers, or web links for publicly available datasets
- A list of figures that have associated raw data
- A description of any restrictions on data availability

The authors declare that the data supporting the findings of this study are available within the paper and supplementary information files.
Source data for figures are provided with the paper.

Field-specific reporting

Please select the one below that is the best fit for your research. If you are not sure, read the appropriate sections before making your selection.

- [x] Life sciences
- [ ] Behavioural & social sciences
- [ ] Ecological, evolutionary & environmental sciences

For a reference copy of the document with all sections, see nature.com/documents/nr-reporting-summary-flat.pdf

Life sciences study design

All studies must disclose on these points even when the disclosure is negative.

Sample size	We did not perform sample-size calculations. Sample size was determined to be adequate based on the magnitude and consistency of measurable differences between groups. For in vitro studies, we basically performed three more independent studies (n larger than or equal to 3).
Data exclusions	Data were only excluded for failed experiments. The reasons for failed experiments included wrong conditions, suboptimal activation and microbial contamination.
Replication	Statistical methods were used to calculate whether similar results were obtained. The experimental findings were reliably reproduced.
Randomization	Samples were randomly allocated into each experimental groups.
Blinding	Investigators were not blinded to different cell types during experiments. Data reported for osteogenesis and adipogenesis studies are not subjective but rather based on quantitative optical absorption.

Reporting for specific materials, systems and methods

We require information from authors about some types of materials, experimental systems and methods used in many studies. Here, indicate whether each material, system or method listed is relevant to your study. If you are not sure if a list item applies to your research, read the appropriate section before selecting a response.

Materials & experimental systems

n/a	Involved in the study	
	x	Antibodies
x		Eukaryotic cell lines
x		Palaeontology and archaeology
	x	Animals and other organisms
x		Human research participants
x		Clinical data
x		Dual use research of concern

Methods

n/a	Involved in the study	
x		ChIP-seq
	x	Flow cytometry
x		MRI-based neuroimaging

Antibodies

Antibodies used	All of the antibodies were purchased from commercial sources with validation data sheets. The information on all antibodies used in the study was listed in Methods section.
Validation	All antibodies validation data sheets were provided by the manufactures.

Animals and other organisms

Policy information about studies involving animals; ARRIVE guidelines recommended for reporting animal research

Laboratory animals	We used genetically modified mice (mus musculus) for this study. Most of the mouse lines have been backcrossed to a C57/BL6 background. Mice with both sexes were used before weaning and male were used after weaned. Mouse strains used in the study were as following: Lepr-cre (JAX008320), Ppp2r1a fl/fl (JAX017441), Col2a1-creERT2 (JAX006774) and Rosa26-CAG-loxP-stop-loxPtdTomato (Ai14: R26R-tdTomato, JAX007914).
Wild animals	N/A
Field-collected samples	N/A
Ethics oversight	These procedures were performed in accordance with protocols (2018-040) approved by the Animal Care and Use Committee in China Medical University and we have complied with all relevant ethical regulations for animal testing and researches.

Note that full information on the approval of the study protocol must also be provided in the manuscript.

Flow Cytometry

Plots

Confirm that:

- [x] The axis labels state the marker and fluorochrome used (e.g. CD4-FITC).
- [x] The axis scales are clearly visible. Include numbers along axes only for bottom left plot of group (a 'group' is an analysis of identical markers).
- [ ] All plots are contour plots with outliers or pseudocolor plots.
- [x] A numerical value for number of cells or percentage (with statistics) is provided.

Methodology

Sample preparation	MSCs were resuspended in 10^6 cells/tube and incubated by Annexin V-FITC and Propidium Iodide at RT for 15 minutes in dark according to the manufacturer's instructions (Strong Biotech Corporation AVK050).
Instrument	BD Accuri™ C6 Plus Flow Cytometer
Software	BD Accuri C6 system
Cell population abundance	Post-sort purity was not determined
Gating strategy	FSC-A vs. SSC-A gates of the starting cell population were used to identify viable cells.

- [ ] Tick this box to confirm that a figure exemplifying the gating strategy is provided in the Supplementary Information.
